# Agitation, Alzheimer’s disease, and autophagy: mechanistic insights into aging pathways, gut microbiome, and artificial intelligence

**DOI:** 10.3389/fimmu.2026.1846280

**Published:** 2026-06-05

**Authors:** Kenneth Maiese

**Affiliations:** Innovation and Commercialization, National Institutes of Health, Bethesda, MD, United States

**Keywords:** Alzheimer’s disease, autophagy, cell senescence, diabetes mellitus, gut microbiota, mechanistic target of rapamycin (mTOR), mood disorders, oxidative stress

## Abstract

The presentation of mood disorders that involve agitation and anxiety in patients with cognitive loss represent significant challenges for the care of patients with Alzheimer’s disease (AD). Additional concerns rest with the rising lifespan and aging of the global population with expectations that over the next two decades more than 50 percent of the elderly population will suffer from mental health disease and at least 30 million of these individuals will also succumb to cognitive loss with AD. Although current treatments for mood disorders and cognitive loss can have a multi-modal approach with behavioral therapy, cognitive training sessions, physical exercise, nutritional care, environmental changes, and disease modifying agents, these therapies are primarily symptomatic in nature that do not halt disease progression and possess risks for further nervous system insults. Given these consideration, novel work that addresses the shared underlying pathways for mood disorders and cognitive loss with autophagy and related mechanisms of programmed cell death, aging and cellular senescence, perivascular system dysfunction, inflammatory microglial cell dynamics, oxidative stress, metabolic pathways that involve diabetes mellitus and apolipoprotein E, the gut microbiota, glucagon-like peptide-1 receptor agonism, innovative diagnostic strategies, artificial intelligence, and machine learning can offer rewarding avenues for the innovative development of therapeutic strategies that address disease onset and progression of these disorders. These pathways that oversee mood disorders and cognitive are both critical and complex in their intimate relationships and warrant in-depth knowledge of the mechanisms that can influence biological outcome for clinical translation.

## 1 Introduction

Mental health disorders impact a significant portion of the global population with over 1 billion individuals, approximately 15 percent of the world’s population, living with psychological disabilities that affect cognitive ability (1–3). The number of individuals affected by mental health can be cyclic and increase such as during periods with viral pandemics (4–8). Depression results in severe sadness, despair, hopelessness, and mood fluctuations that leads to the loss of memory function that affects over 300 million people. Frequency of depression occurs approximately 50 percent more in women than in men. Although depression risk is highest over the age of 65, it is important to note that suicide is the 4^th^ greatest cause of death from 15 to 29 years old (9). In addition, individuals with developmental and intellectual disabilities that affect cognition also have an increased rate of mental health conditions, such as depression (10). Anxiety and agitation also result in cognitive loss with approximately 400 million individuals suffering from excessive motor activity, irritability, hostility, impulsiveness, destructive behavior, mental tension, inability to have mental focus, and fear. In the body, depression, anxiety, and agitation can result in sleep disturbances, rapid and irregular heart rate, muscle contractions, hyperhidrosis, fatigue, restlessness, and gastrointestinal distress. Given that these behavioral health disorders severely impact the brain, recent work suggests that disorders such as anxiety and agitation can lead to chronic neurodegenerative disease as well, such as with Alzheimer’s disease (AD) (11–15). In addition, with demyelinating disease and the early stages of multiple sclerosis (MS), at least 30 percent of patients also have depression (15, 16). Disorders of behavior can affect multiple pathways in the nervous system such as exacerbating sleep fragmentation, circadian rhythm and clock dysregulation, impairment in perivascular system function, neuroinflammation, and mitochondrial dysfunction that ultimately lead to cognitive loss and dementia (13, 17–25) (**Table 1**).

**Table 1:** Highlights.

Agitation, alzheimer’s disease, and autophagy: mechanistic insights into aging pathways, gut microbiome, and artificial intelligence
• Mood disorders that include agitation and anxiety affect at least 15 percent of the global population and severely impact the brain leading to cognitive loss in diseases with Alzheimer’s disease (AD) and multiple sclerosis (MS).
• With the increase in global lifespan and the aging population over the next two decades, over 2 billion people will be greater than 60 years old and at least 30 million individuals will have AD with 85 percent of these patients presenting with mood disorders.
• Treatment of mood disorders that occur in AD and MS patients represent formidable clinical challenges and though current treatments for mood disorders are broad in scope, they remain symptomatic in nature, do not halt disease progression, and can lead to unwanted clinical outcomes that include brain edema and microhemorrhages.
• Given these challenges, innovative strategies are highly warranted that can address the shared underlying pathways that link mood disorders and cognitive loss together involving autophagy and related mechanisms of programmed cell death, aging and cellular senescence, perivascular system dysfunction, inflammatory microglial cell dynamics, oxidative stress, metabolic pathways that involve diabetes mellitus and apolipoprotein E (APOE), the gut microbiota, glucagon-like peptide-1 (GLP-1) receptor agonism, innovative diagnostic strategies, artificial intelligence, and machine learning.
• These shared underlying pathways intersect with several mechanistic pathways and entities that include transient receptor potential cation channel subfamily V member 1 (TRPV1) family receptors, ferroptosis, pyroptosis, short-chain fatty acids with gut microbiota, gamma-aminobutyric acid (GABA) neurotransmission, protein kinase B (Akt), the mechanistic target of rapamycin (mTOR), triggering receptor expressed on myeloid cells 2 (TREM2), severe acute respiratory syndrome (SARS-CoV-2), coronavirus disease 2019 (COVID-19), and long-COVID-19 infection, and neurofilament light chain (NFL).
• These pathways offer exciting prospects for the development of new strategies that can treat disease onset and progression for mood disorders and cognitive loss, but they must be recognized as complex in nature and intimately connected with one another that necessitates fine modulation of these pathways through regulatory mechanisms, such as with the mammalian target of rapamycin (mTOR), to yield efficacious outcomes for clinical translation.

Behavior disorders are intimately tied to the clinical presentations of cognitive diseases, such as AD and MS. Agitation along with anxiety are formidable challenges in caring for patients with AD and can be present in more than 85 percent of patients with AD, especially during the later stages of cognitive impairment (12, 26, 27). In patients with MS, agitation and anxiety present 3 time greater than the general population rate such that almost 40 percent of patients with MS suffer from mood disorders (1, 15, 16, 28).

**Figure 1:**
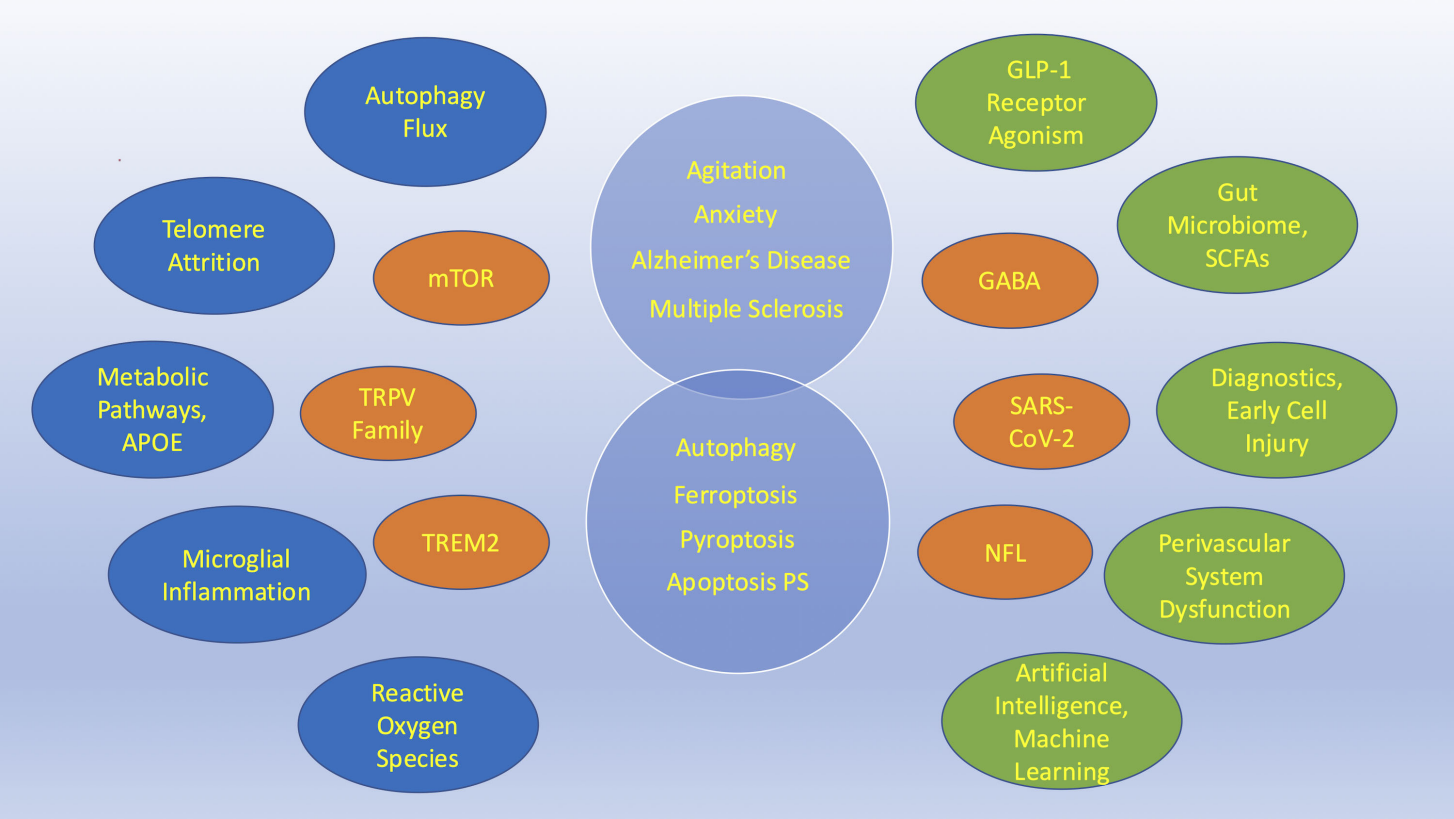
Elucidating the underlying shared pathways that bind mood disorders and cognitive loss together. Innovative investigations are highly needed to address the shared underlying pathways of these disorders for the development of novel strategies to treat both disease onset and disease progression. Autophagy and related mechanisms of programmed cell death that involve ferroptosis, pyroptosis, and apoptosis with phosphatidylserine (PS) membrane externalization are critical pathways for mood disorders and cognitive loss. Ultimately they are intimately connected to multiple cellular mechanisms and entities that oversee aging with telomere attrition, perivascular system dysfunction, transient receptor potential cation channel subfamily V (TRPV) family members, microglial cell inflammation, reactive oxygen species, metabolic pathways with apolipoprotein E (APOE), gamma-aminobutyric acid (GABA) neurotransmission, gut microbiome with modulation of glucagon-like peptide-1 (GLP-1) receptor agonism and short-chain fatty acids (SCFAs), autophagy flux mechanisms such as with the mechanistic target of rapamycin (mTOR), triggering receptor expressed on myeloid cells 2 (TREM2), severe acute respiratory syndrome (SARS-CoV-2), diagnostics such as with neurofilament light chain (NFL) to detect early cell injury, artificial intelligence, and machine learning applications. In the image, the dark blue left side circles represent cellular systems, such as autophagy flux and reactive oxygen species, that can influence clinical outcomes for mood disorders and cognitive loss. The green right side circles represent tissue and endocrine systems, such as the gut microbiome and GLP-1, and diagnostic pathways that affect mood disorders and cognitive loss. The small orange circles represent underlying shared pathways for mood disorders and cognitive loss, such as mTOR and GABA pathways.

The origin of agitation and anxiety can result from multiple systems in the brain, but the prevalence of these mental disorders is rising with the advancing age of the global population and affecting the most susceptible individuals with intellectual disability and dementia (10). The lifespan and age of the world’s population is increasing in developed and developing nations with the expectation that almost 20 percent of individuals will be living beyond the age of 60 in the year 2030 (29–31). By the year 2025, over 2 billion people will be greater than 60 years old and more than 425 million people will attain the age of 80 or older (32–35). With the rise in lifespan, a corresponding increase in clinical mental health disorders and behavior impairment has occurred as a result of aging processes involving cellular senescence. In fact, in the year 2050, almost 50 percent of individuals who reach the age of 75 are expected to suffer from mental health disease (36). Cellular senescence is overseen by telomere (TL) processing that maintains genomic deoxyribonucleic acid (DNA) to modulate cell replication and cell survival (37–39). With progressive aging, the function of TLs is lost and cellular senescence leads to failed cellular replication, cell death, oxidative stress, loss of metabolic homeostasis, mitochondrial injury, alterations in the gut microbiome, and programmed cell death (31, 40–44) (**Figure 1**).

The underlying pathways associated with aging and cellular senescence also can be responsible for cognitive loss with disorders such as AD and MS. Familial AD (FAD) is a rare condition that involves approximately 200 families throughout the world and occurs before the age of 55. FAD is the result of amyloid precursor protein (APP) gene mutations that are present on chromosomes 1, 14, and 21 and lead to memory loss (45–48). The sporadic form of AD affects the majority of individuals over the age of 65, is the most common form of dementia, and is believed to represent at least 70 percent of cases. Currently at least 7 million individuals in the United States (US) have AD and this is expected to grow in number to 30 million individuals over the next two and a half decades (49–53). Additional disorders in the nervous system that also lead to cognitive loss, such as MS, result in word finding difficulties and impairments with object naming with women more often being afflicted with MS than men (15, 28, 54–57). Approximately 65 percent of people with MS experience loss of executive functions that involve information recall, memory, and attention disorders (1, 42, 58–60). Telomere attrition can be a factor in the progression of mood disorders, AD, and MS with the production of oxidative stress that leads to neuronal and vascular cell loss and can function synergistically with lower socioeconomic status (61–63) (**Table 1**).

Interestingly, the perivascular system of the brain can affect mental health disease and cognitive loss disorders (20, 64) (**Figure 1**). The perivascular system is the brain’s mechanism for removal of waste that involves a perivascular network with cerebrospinal fluid. Relying upon arterial pulsing and astrocytic cells, the perivascular system removes metabolic waste, inflammatory mediators, and toxins that can include β-amyloid (Aβ), tau, and α-synuclein (20, 22, 65, 66). Perivascular system impairment in behavior disorders, such as with agitation and anxiety, leads to inflammation, oxidative stress, circadian clock disturbances, sleep disruption, and cognitive decline (66, 67). During AD and memory loss, the perivascular system can become dysfunctional that promotes Aβ and tau deposition, sleep fragmentation, and brain edema and hemorrhage with amyloid-related imaging abnormalities following immunotherapy treatments for AD (68). Loss of sleep and sleep fragmentation critically impacts circadian rhythm function and prevents the removal of brain toxins through the perivascular system to result in cognitive loss (22, 25, 34, 69, 70). Sleep loss in conjunction with perivascular system dysfunction also can lead to cognitive loss and susceptibility to infection with severe acute respiratory syndrome coronavirus 2 (SARS-CoV-2), coronavirus disease 2019 (COVID-19), and long-COVID-19 infection, also known as long-haul COVID, chronic COVID-19, or post-acute COVID (5, 8, 71–73). During periods of metabolic distress with diabetes mellitus (DM) and cardiac insufficiency, failure of the perivascular system leads to memory impairment, anxiety, agitation, increased Aβ and tau deposition, circadian impairments, gut dysbiosis, and programmed cell death with autophagy (46, 74–78) (**Table 1**).

## 2 Autophagy, oxidative stress, and metabolic dysfunction

Programmed cell death pathways that involve autophagy are an intricate part of the processes that oversee behavior disorders and cognitive loss (79–85). Autophagy involves the engulfment of organelles and subcomponents in the cytoplasm that can be recycled for later use by cell (86–89). Three types of autophagy exist that consist of macroautophagy, microautophagy, and chaperone-mediated autophagy with the pathways of macroautophagy most commonly described. Macroautophagy relies upon autophagosomes that combines cytoplasmic proteins and organelles for degradation through the use of lysosomes, microautophagy also employs lysosomes but through invagination of lysosome membranes for the recycling of proteins, and chaperone-mediated autophagy processes lysosomes for degradation of organelles through the transfer of “protein chaperones”.

In mood disorders, autophagy can become dysregulated, lead to the release of inflammatory cytokines, and promote depressive behavior. Restoration of autophagy induction blocks mood disturbances (9). In experimental studies of social interaction and cognitive performance, autophagy activation through triggering receptor expressed on myeloid cells 2 (TREM2) can improve memory and behavior function (81). TREM2 also can modulate microglial cell polarization, reduce inflammation, and limit oxidative stress to increase cell survival (33, 90–92). Microglia are critical modulators of cellular inflammation, autoimmunity, and oxidative stress that influence overall cognitive function (93–97). Microglia that have increased autophagic flux in the prefrontal cortex can promote transcription of autophagy-related genes Atg6, Atg7, and Atg12 that alleviate social avoidance, agitation, and stress-induced behavioral changes (83). Autophagy activation is modulated through an inverse relationship with the mechanistic target of rapamycin (mTOR). The blockade of necessary autophagy pathways by mTOR can promote social dysfunction, anxiety, and agitation (79, 80, 98, 99). In disorders with agitation and anxiety, such as autism spectrum disorder, excessive mTOR function also can limit autophagy pathways that are required to control inhibitory and excitatory neuronal pathways (100, 101) (**Table 1**).

During experimental studies with cognitive impairment and AD, activation of autophagy can reduce oxidative stress, decrease Aβ deposition, reduce phosphorylation and activity of protein kinase B (Akt) and mTOR, and enhance learning and memory performance (102–104). Reductions in autophagy flux that may be overwhelmed by oxidative stress can lead to accelerated brain aging and decreases in memory performance (105). Autophagy activation that limits autophagosome accumulation provides cellular protection against Aβ toxicity (106). Increased activation of the transient receptor potential cation channel subfamily V member 2 (TRPV2) uses autophagy as an indispensable mechanism to diminish neuroinflammation, improve mitochondrial function, and block Aβ cell death (50, 107, 108). Related to TRPV2 channel activation, the TRPV1 family receptors also can lead to autophagy induction to result in neuroprotection, alleviate behavioral dysfunction, and block oxidative stress in the brain (109–112). Activation of autophagy can suppress APP cleavage enzyme 1 and foster Aβ clearance in animal models of AD (113). In experimental models of MS, autophagy can reduce clinical symptoms, cytokine release, and modulate microglial activity to delay disease progression (15, 114, 115) (**Figure 1**).

Of note, other pathways of programmed cell death that pertain to ferroptosis and pyroptosis may work in conjunction with autophagy pathways with behavioral disorders and cognitive loss. Ferroptosis is a result of excess iron storage in cells that leads to lipid peroxidation, oxidative stress, and glutathione homeostasis loss (42, 116, 117). Ferroptosis plays a role in neurodegeneration with behavior disturbances and cognitive loss (42, 69), neuronal cell death with epilepsy (118), and motoneuron impairment with memory loss (90). Pyroptosis is controlled by the inflammasome family of nucleotide-binding oligomerization domain and leucine-rich repeat-containing receptors (NLRs) that contains NLRP1, NLRP3, NLRP6, and NLRC4. The inflammasome, also known as the pyroptosome, through the NLRs gasdermin proteins leads to caspase 1, caspase 4, and caspase 5 activation and the release of cytokines (119–123). Pyroptosis is responsible for microglial polarization and pro-inflammatory cytokine release that leads to tau pathology, neuronal cell death, and cognitive loss (90, 123). During treatment with disease-modifying therapies (DMT) with MS, pyroptosis can occur in patients and may function as a response biomarker (124). Autophagy, ferroptosis, and pyroptosis also may function in concert to foster mood disorders and cognitive loss. For example, autophagy, ferroptosis, and pyroptosis can lead to cell demise during cell ischemia, toxic cell environments, and inflammation to promote agitation, anxiety, and cognitive loss (42, 69, 125–127).

Both oxidative stress and disorders of cellular metabolism that involve DM are critical components of the autophagy pathways (40, 105, 128–135). Oxidative stress occurs as the result of reactive oxygen species (ROS) generation that involve free radicals with superoxide free radicals, nitric oxide, singlet oxygen, peroxynitrite, and hydrogen peroxide that lead to cellular injury (136–141). In mood disorders, oxidative stress can further aging and circadian rhythm dysfunction (17), perivascular system impairment (20), cytoskeletal and metabolic pathway dysregulation (21), disruption of gamma-aminobutyric acid (GABA) connections (142), reduction of glial cells in brain white matter (143), diminish mitochondria-related gene expression (117, 144), decrease neuronal cell survival (144, 145), and injure myelinated fibers (27). During cognitive loss with AD and MS, oxidative stress leads to similar detriments as with mood disorders of agitation and anxiety to result in deposition of Aβ and tau in the brain (146), loss of cortical and deep gray matter in the brain (142, 147), neuronal optic tract degeneration with demyelination (114), glial and neuronal cell death (148), alterations in glutamatergic signaling in the brain that also may contribute to delirium (149, 150), mitochondrial injury (52, 151–153), and activation of microglial neuroinflammatory pathways (48, 93). Oxidative stress also is a significant component of underlying metabolic dysfunction that involves DM as a co-morbidity with mood disorders and cognitive impairment. Oxidative stress with DM results in depressed mood, cortical brain injury, and vasculature dysfunction (154, 155), anxiety with depressed insulin like growth factor-1 levels and diminished superoxide dismutase enzyme activity (145); increased neurofilament light chain (NFL) serum levels that are diagnostic of developing brain injury (156–158), reduced insulin sensitivity that results in cognitive dysfunction (159–161), cellular calcium overload and synaptic cell loss (162), brain Aβ accumulation (163), aging with cellular senescence (134, 164); spatial memory loss with hippocampal dysfunction (165); and vascular disease with retinopathy (166) (**Figure 1**).

The generation of ROS and oxidative stress may play a dual role in the body. Under normal physiological conditions, antioxidant systems that include catalase, superoxide dismutase enzyme, glutathione peroxidase, and vitamins C, D, B, E, and K are in place to limit the detrimental effects of prevent oxidative stress (44, 99, 133, 167–170). However, even during scenarios that lead to ROS generation, oxidative stress may not be detrimental or only lead to cellular injury under specific environmental circumstances. In this regard, oxidative stress may not alter the cellular redox balance to lead to cell death but rather promote immune homeostasis pathways with microglial activity that can assist with toxic environments. These potential beneficial effects of ROS appear to be closely controlled by energy depletion pathways with the coenzyme ß-nicotinamide adenine dinucleotide (NAD^+^), be sex specific, and only affect susceptible cells and tissues (34, 93, 130, 171, 172).

As part of the cellular metabolic pathways associated with agitation, anxiety, and memory disorders, apolipoprotein E (APOE) can play a significant role (**Figure 1**). APOE can significantly influence the course of mood disorders and cognitive loss with AD as well as MS (5, 15, 173–175). Produced in the liver by hepatic cells, APOE oversees lipid metabolism through cellular transport of phospholipids, triglycerides, and cholesterol (5, 120, 173, 175, 176). In the nervous system, APOE is generated in astrocytes to transfer cholesterol to neurons through receptors of APOE (164, 174, 177, 178). In mood disorders, the apolipoprotein E (APOE-ϵ4) allele can be associated with moderate or severe depression, symptoms of anxiety, and cognitive impairment (5). Experimental studies further support these observations since the ϵ4 allele of the apolipoprotein E (*APOE-ϵ4*) gene is associated with increased anxiety in murine models and in clinical trials patients with two *APOE-ϵ4* alleles suffer from increased anxiety scores (179). In regard to memory loss, individuals that are homozygous with allele *ϵ4/ϵ4* have an increased risk of developing AD more than 20 times than individuals without these alleles by the age of 85 (120, 175, 180). Individuals that are either homozygotes and heterozygotes with APOE-ϵ4 carrier status have the risk of accelerated age-related cognitive loss after 70 years of age (181). APOE-ϵ4 carrier status is associated with cognitive loss in MS patients that is reflected by slower responses to stimuli (174). In addition, the *APOE* ϵ3/ϵ3 genotype can raise the risk for males to develop optic neuritis (173). Other considerations with APOE-ϵ4 carrier status reveal that individuals are more susceptible to infection with SARS-CoV-2 and COVID-19 that leads to long-COVID dementia, brain microhemorrhages, anxiety, and depression in patients with AD and MS (5, 15, 73, 176, 182, 183). At the cellular level, APOE-ϵ4 carrier status is associated with impaired autophagy function, endoplasmic reticulum stress, altered mTOR signaling, and dysfunctional mitochondrial bioenergetics (180, 184). On the converse side, *APOE-ϵ2* alleles can be protective with diminished deposition of Aβ aggregation and reduction in apoptotic phosphatidylserine (PS) membrane exposure and cell death (185, 186) (**Table 1**).

## 3 The gut microbiome, novel diagnostics, and artificial intelligence

Given the pathways of autophagy, oxidative stress, and cellular metabolism that can tie underlying pathologies of mood disorders and cognitive loss together, systems exclusive of the nervous system that may not be immediately apparent can significantly influence these disorders as well, especially those involving the gut microbiota (187–192). Developed from birth with changes occurring into adulthood, the gut microbiota is also known as the gut flora or the gut microbiome and contains fungi, viruses, bacteria, and archaea in the digestive tract (31, 187, 193). The gut microbiota functions as an organ of the endocrine system containing 100 billion bacteria that can generate vitamins, such as vitamin B, and produce short-chain fatty acids (SCFAs) that are beneficial to the body (49, 190, 193, 194). Direct therapeutic changes to the gut microbiota, such as with Lactobacillus growth, altering the ratio of the Bacteroidota and Firmicutes species, and promoting mTOR activation, can increase SCFAs production to improve metabolic function and reduce inflammation for mood disorders and cognitive function (193–199). Changes to the gut microbiome can reduce symptoms of anxiety and depression (195), limit Aβ aggregation and oxidative stress (49), diminish inflammatory cytokines that can prevent hippocampal degeneration in models of AD (196); and control indole metabolites, such as 3-indollepropionic acid (IPA), to block tau phosphorylation for improved memory (13, 200).

The production of SCFAs in the digestive tract can lead to the generation and secretion of glucagon-like peptide-1 (GLP-1) (201) (**Figure 1**). GLP-1 receptor agonism can influence mood disorders and memory impairment that are mediated by metabolic pathways. Through GLP-1 receptor agonism pathways, new treatments that involve semaglutide, tirzepatide, and liraglutide to treat metabolic disorders for glucose homeostasis and obesity may have overlap for applications to address mood disorders and cognitive loss (50, 82, 202–205). GLP-1 receptor agonism can resolve depressive-like phenotypes and limit cognitive loss (82), maintain mitochondrial integrity and GABA transmission (205), improve cognition with a decrease in insulin resistance (206), offer protection against metastatic brain disease during DM (207), and may limit cortical brain injury as suggested with reductions in NFL brain immunostaining that are indicative of neuronal cell death (208). GLP-1 receptor agonists are now also employed for nonalcoholic fatty liver disease, also known as metabolic dysfunction-associated steatotic liver disease (50, 209). Such applications may be relevant since recent work suggests that liver-derived exercise factor (exerkine) may reduce the effects of aging on memory with AD through protection of the brain vasculature (210). Activation of GLP-1 receptors also can work in conjunction with TRPV1 receptors and be beneficial for mitochondrial dynamics, reduction in oxidative stress, protection of pancreatic β cells, and maintenance of insulin receptor function (50, 211–214) (**Table 1**).

The cellular pathways of GLP-1 agonism are reliant on the pathways of autophagy. Under some conditions, GLP-1 receptor agonism uses autophagy to protect musculoskeletal cells during inflammation (215) and neuronal retinal cells during DM retinopathy (216). In other scenarios, GLP-1 agonism limits autophagy activation and employs Akt and mTOR activation for neuronal cell protection during elevated glucose and toxic environments (205, 217, 218), maintenance of glucose homeostasis for prevention of cognitive loss and depression (82), and for the preservation of pancreatic β-cells during cholesterol-mediated apoptosis (212, 219). These observations point to the critical modulation of cellular autophagy flux through the inverse relationship with mTOR, since exacerbated mTOR activity or the presence of ineffective autophagy induction can lead to detrimental biological outcomes with GLP-1 receptor agonism. Such an imbalance in cellular autophagy flux levels may have a role in the emerging unwanted clinical effects seen with GLP-1 receptor treatments that involve acute pancreatitis, anterior ischemic optic neuropathy, nausea, depression, alopecia, vomiting, and diarrhea (220–222).

The employment of novel diagnostics can further foster the implementation of new care pathways that are founded on the common underlying pathways shared between mood disorders and cognitive loss (157, 223–225) (**Figure 1**). Although the clinical examination will always be the foundation for patient care, early detection of the development of mood disorders and cognitive loss may assist in limiting irreversible damage to the brain. For example, programmed cell death with apoptosis has two distinct phases. The first phase that involves membrane PS residue externalization and inflammatory microglia that can remove injured cells with PS externalization. Microglia can have dual role in the brain by eliminating Aβ deposits in the brain (86, 97, 226–228), but at times microglia also may foster cellular environments with oxidative stress (48, 93, 95, 229). Yet, the first phase of apoptosis is reversible and if recognized early, PS membrane externalized can be corrected such that microglia do not interfere with functioning cells and cell death is prevented (84, 104, 230–235). If the second phase of apoptosis is reached, this becomes an irreversible process with mitochondrial dysfunction, oxidative stress, caspase activation, and DNA degradation (133, 236–240). As a result, diagnostic biomarkers for mood disorders and memory impairment can offer the ability to detect cellular disease processes early that may allow interventions to prevent cell injury, mood disorders, and cognitive impairment. Innovative diagnostics that can be delivered through point-of-care applications can rely upon the detection of circular ribonucleic acids (circRNAs) to identify endothelial cell dysfunction and atherosclerosis for impaired cerebral blood flow that can affect agitation, anxiety, and cognition (241–243). Early pathology detection that is associated with ischemic brain disease may be detected through pathways that oversee mTOR and programmed cell death with pyroptosis (244). Dysregulation in circadian rhythms and clock genes, especially in the context of sleep disorders can involve diagnostics with exosomes as early detection of cognitive loss (224, 245). Loss of mammalian forkhead transcription factors, such as FoxO1 in the brain, may signal early events for Aβ deposition and the development of AD (246). NFL serum levels that are elevated also may be a biomarker that signals years in advance the death of neurons and chronic injury to the brain that leads to mood disorders, AD, and MS cognitive loss (60, 158, 247, 248). Biomarkers also may be employed as clinical response markers for treatment, such as in MS, to detect proinflammatory cytokine release that can impair cognition (124).

Knowledge gained through the shared pathways of mood disorders and cognitive loss as well as the contribution of the gut microbiota and the use of novel diagnostics can present challenges to properly synthesize this vast amount of data into usable information for clinical care (**Table 1**). Strategies that can incorporate the use of artificial intelligence (AI) and machine learning (ML) can meet these challenges for the compilation of significant information for the development of new treatments for agitation, anxiety, and memory impairment. AI and ML are processes that are capable of evaluating enormous data arrays that originate from broad non-homogenous parameters such as cellular pathways, genetic information, pathological tissue, liquid biopsies, and imaging. This data through AI and ML are assessed and synthesized to provide predictive analysis for signature clinical pathways that are not available from the singular use of statistical methods alone (30, 152, 157, 223, 249–251). Recent studies demonstrate that models with AI that use 50 predictors, including medical and psychiatric histories, for the assessment of clinical agitation can detect the risk for the development of agitation in patients as well as the important co-morbidities that pertain to altered cognition (252). With anxiety and depression, ML models can detect mood disorders with high accuracy to serve as decision-support tools for physicians, but these models require further refinement with potential incorporation of additional data sets from voice recordings and physiological wearables (253). Through the AI analysis of genetic transcriptional states, cellular metabolism, inflammatory mediators, and lipid pathways, multiple genes that can confer risk for AD, mood impairment, memory loss, and microglial alterations can be identified (254, 255). ML algorithms have noted dysregulation in multiple plasma proteins that can lead to dementia and loss of longevity that involve autophagy pathways, monocyte depletion, nucleosome assembly, and oxidative stress (33, 256–258). Studies that use AI analysis with brain-region and cell-specific signatures suggest that microglial cell, gut dysbiosis, APOE risk, programmed cell death, and gene transcription dysregulation can affect mood disorder dysfunction and lead to AD progression (259). The studies with AI and ML further highlight the intimacy of the shared pathways for mood disorders and AD that can lead to the onset and progression of these diseases (**Figure 1**).

## 4 Discussion

Mood disorders that involve agitation, anxiety, and depression, affect over 1 billion individuals throughout the globe. These disorders lead to symptoms of excessive motor activity, irritability, hostility, impulsiveness, destructive behavior, loss of attention, and fear that ultimately are associated with the onset of neurodegenerative disorders that involve cognitive loss with AD and MS. Mood disorders that involve agitation and anxiety represent significant challenges for the treatment of patients with AD and MS with greater than 85 percent of AD patients and almost 40 percent of individuals with MS suffering from agitation and anxiety. Additional concerns for the clinical care of mood disorders and cognitive impairment rest with the increased lifespan and aging of the global population with expectations that over the next two decades more than 2 billion people will be greater than 60 years old, approximately half of the population at 75 years of age may suffer from mental health disorders, and over 30 million individuals will have cognitive loss with AD.

Current therapies for mood disorders and cognitive loss are principally symptomatic in nature and warrant novel considerations for the development of strategies that can address underlying shared pathways for effective clinical care. Treatments for mood disorders, such as for agitation and anxiety, involve a combined approach with antipsychotics, promotion of GABA neurotransmission, selective serotonin reuptake inhibitors, serotonin-norepinephrine reuptake inhibitors, behavioral therapy, environmental changes, lifestyle alterations, nutritional care, physical activity, and structured support groups, but disease progression continues even if the course is cyclic in nature (13, 23, 260). In regard to memory loss with AD and MS, therapies are also multi-modal in scope to include cholinesterase inhibitors, cognitive training sessions, physical exercise, monitoring with wearable biosensors, disease modifying therapies (DMTs) for MS to limit relapsing–remitting episodes of MS, and immunotherapy agents designed to reduce Aβ deposition in the brain, but these treatment also cannot prevent the progression of the disease. Furthermore, additional challenges exist since recently approved US Food and Drug Administration treatments with brexpiprazole that seek to treat mood disorders in AD can only offer symptomatic care, brain immunotherapy agents for AD have the risk of edema and cerebral microhemorrhages, and cognitive impairment in MS continues with DMTs despite limits in brain volume loss (15, 261–263). As a result, innovative studies that involve pathways of autophagy and related mechanisms of programmed cell death, aging with cellular senescence, perivascular system dysfunction, inflammatory microglial cell dynamics, oxidative stress, metabolic pathways that involve DM and apolipoprotein E (APOE), the gut microbiota, glucagon-like peptide-1 (GLP-1) receptor agonism, novel diagnostic strategies, and AI offer fruitful opportunities to dissect the intimately shared cellular mechanisms of mood disorders and cognitive loss for the development of care pathways that can address the onset and progression of these disorders.

It is important to note that these shared underlying pathways are intimately reliant upon one another and continual understanding of the mechanisms that control these pathways is critical for effective translation to clinical care for mood disorders and memory loss. Loss of cellular metabolic homeostasis, such as with DM as a co-morbidity, can significantly influence the pathways that bind mood disorders and cognitive loss together since metabolic distress can promote conditions that lead to failure of the perivascular system resulting in agitation, anxiety, cognitive loss, deposition of Aβ and tau, circadian clock gene dysfunction, gut dysbiosis, susceptibility to SARS-CoV-2 and COVID-19 brain infection, and autophagy flux changes. In the metabolic pathway, APOE also influences the course of agitation, anxiety, and memory loss but may have variable influences on the progression of disease pathology. With patients that are homozygous with *APOE-ϵ4* alleles, multiple conditions can ensure that include increased anxiety scores, depression, accelerated age-related cognitive loss, increased risk of developing AD, slower responses to stimuli in patients with MS, and a higher risk for long-COVID dementia. However, *APOE-ϵ2* alleles can offer significant protection during mood disorders and cognitive loss with reduction in apoptotic PS membrane exposure and cell death. In regard to autophagy, activation of this programmed cell death pathway can improve memory and behavior function, oversee excitatory neuronal pathways in mood disorders through microglial modulation, reduce oxidative stress, decrease Aβ deposition, maintain mitochondrial function, limit aging processes, and control cytokine release. Yet, autophagy at times can work in conjunction with ferroptosis, and pyroptosis to result in cell death, toxic cell environments, and inflammation to foster agitation, anxiety, and cognitive loss. In addition, increased autophagy levels can lead to oxidative stress and endoplasmic reticulum stress, mitochondrial dysfunction, viral antigen infection, depression, and cognitive loss (160, 264, 265). Under some conditions, GLP-1 agonism requires reduction in autophagy levels with the activation of mTOR to maintain glucose homeostasis, protect neuronal cells, and preserve pancreatic β-cells during cholesterol-mediated apoptosis. Pathways with mTOR are vital to maintaining proper levels of autophagy flux to reach desired biological outcomes, since conditions with either excessive or insufficient autophagy activation can result in clinical presentations of agitation, anxiety, and cognitive loss. Further insights into the intricate relationships of theses shared underlying pathways will be invaluable for the AI assessment of varied cellular pathways and other parameters that tie mood disorders and cognitive loss together and for the ultimate development of clinical strategies for these diseases.
